# One-to-One and Group-Based Teleconferencing for Falls Rehabilitation: Usability, Acceptability, and Feasibility Study

**DOI:** 10.2196/19690

**Published:** 2021-01-12

**Authors:** Helen Hawley-Hague, Carlo Tacconi, Sabato Mellone, Ellen Martinez, Lorenzo Chiari, Jorunn Helbostad, Chris Todd

**Affiliations:** 1 School of Health Sciences University of Manchester Manchester United Kingdom; 2 Manchester Academic Health Science Centre Manchester United Kingdom; 3 Interdepartmental Center for Industrial Research Health Sciences and Technologies University of Bologna Bologna Italy; 4 mHealth Technologies s.r.l. Bologna Italy; 5 Department of Electrical, Electronic and Information Engineering "Guglielmo Marconi" University of Bologna Bologna Italy; 6 Manchester University NHS Foundation Trust Manchester United Kingdom; 7 Department of Neuromedicine and Movement Science The Faculty of Medicine and Health Sciences Norwegian University of Science and Technology, NTNU Trondheim Norway

**Keywords:** aged, postural balance, telerehabilitation, patient compliance, accidental falls, mobile phone

## Abstract

**Background:**

Falls have implications for the health of older adults. Strength and balance interventions significantly reduce the risk of falls; however, patients seldom perform the dose of exercise that is required based on evidence. Health professionals play an important role in supporting older adults as they perform and progress in their exercises. Teleconferencing could enable health professionals to support patients more frequently, which is important in exercise behavior.

**Objective:**

This study aims to examine the overall concept and acceptability of teleconferencing for the delivery of falls rehabilitation with health care professionals and older adults and to examine the usability, acceptability, and feasibility of teleconferencing delivery with health care professionals and patients.

**Methods:**

There were 2 stages to the research: patient and public involvement workshops and usability and feasibility testing. A total of 2 workshops were conducted, one with 5 health care professionals and the other with 8 older adults from a community strength and balance exercise group. For usability and feasibility testing, we tested teleconferencing both one-to-one and in small groups on a smartphone with one falls service and their patients for 3 weeks. Semistructured interviews and focus groups were used to explore acceptability, usability, and feasibility. Focus groups were conducted with the service that used teleconferencing with patients and 2 other services that received only a demonstration of how teleconferencing works. Qualitative data were analyzed using the framework approach.

**Results:**

In the workshops, the health care professionals thought that teleconferencing provided an opportunity to save travel time. Older adults thought that it could enable increased support. Safety is of key importance, and delivery needs to be carefully considered. Both older adults and health care professionals felt that it was important that technology did not eliminate face-to-face contact. There were concerns from older adults about the intrusiveness of technology. For the usability and feasibility testing, 7 patients and 3 health care professionals participated, with interviews conducted with 6 patients and a focus group with the health care team. Two additional teams (8 health professionals) took part in a demonstration and focus group. Barriers and facilitators were identified, with 5 barriers around reliability due to poor connectivity, cost of connectivity, safety concerns linked to positioning of equipment and connectivity, intrusiveness of technology, and resistance to group teleconferencing. Two facilitators focused on the positive benefits of increased support and monitoring and positive solutions for future improvements.

**Conclusions:**

Teleconferencing as a way of delivering fall prevention interventions can be acceptable to older adults, patients, and health care professionals if it works effectively. Connectivity, where there is no Wi-Fi provision, is one of the largest issues. Therefore, local infrastructure needs to be improved. A larger usability study is required to establish whether better equipment for delivery improves usability.

## Introduction

### Background

There are approximately 55,000 falls-related emergency hospital admissions in England among patients aged 65 years and older, and around a third of people aged 65 years and above fall each year [1], costing the National Health Service (NHS) £4.6 million (US $6.2 million) per day [1]. Strength and balance exercises have been proven to be effective in reducing the risk and rate of falls [2-4]. However, for these exercises to be effective, a minimum effective dose (3 times a week) must be reached and then maintained in the long term for sustained effects [3]. We know that the role of health care professionals is important in both motivating older adults and progressing their exercise to ensure that the exercises are challenging [5,6]. Currently, strength and balance programs delivered by NHS falls rehabilitation services are inadequate in dose [7], and most services see patients only once per week [7].

Teleconferencing could be an effective way of delivering evidence-based strength and balance exercises by providing increased contact with health care professionals. It has been demonstrated that introducing video consultations is complex and disrupts established processes and routines [8,9]. Concerns have been raised about technical and clinical quality, privacy, safety, and accountability [8,9]. The evidence base on remote consultations by video technology is increasing [10-12], and studies have reported positive benefits and similar satisfaction levels. However, studies that focus on the role of teleconferencing for fall prevention are sparse. Some studies have focused on the delivery of Tai Chi [13,14], and others have focused on other types of rehabilitation [15-17]. The systematic review by Kairy et al [15] examines the clinical outcomes, clinical process, health care utilization, and costs associated with telerehabilitation (therapy delivered through teleconferencing). Clinical outcomes of telerehabilitation programs were found to be as good if not better when compared with those of standard programs. In addition, adherence to telerehabilitation was found to be good. Other reviews have provided some potential but are still not conclusive [18]. Social networks and friendship have also been identified as important aspects of group telerehabilitation programs [19].

As health professionals do not need to travel (cost and time) to patients’ houses, teleconferencing could allow them to see patients more regularly than once a week, increasing exercise dose and motivation. We know that health professionals are an important source of motivation [5,6,20]. Strength and balance exercises could be delivered both one-to-one in patients’ homes or in groups. Some patients do not have the confidence or ability to attend face-to-face group exercise sessions [21]. It may be that being part of a small virtual group either increases confidence and ability to attend a face-to-face group session or enables adherence through long-term exercise and peer support available in the home [22].

### Objectives

The aim of our study is to examine whether smartphone-based teleconferencing (linked to a television [TV] or screen) is usable, acceptable, and feasible for health professionals and older adults as a means of delivering evidence-based fall prevention strength and balance home exercise programs. Acceptability is a multifaceted construct that considers the extent to which people that deliver or receive a health care intervention consider it to be appropriate [23]. When referring to feasibility, we particularly focus on practicality (to what extent teleconferencing could be conducted with the intended participants using existing means, resources, and circumstances) and implementation (to what extent teleconferencing could be used to successfully deliver rehabilitation to intended participants) [24]. Usability focuses on whether a person can use it for its intended purpose [25]. Models such as the technology acceptance model (TAM), which focuses on whether a technology is perceived as useful and whether it is easy to use [26], are important when developing technological interventions and are considered within our study. We took a two-step approach to explore acceptability, usability, and feasibility.

## Methods

### Patient and Public Involvement Workshops

We held 2 patient and public involvement (PPI) workshops to gain initial feedback on teleconferencing:

1. Group of health professionals from a Manchester Falls Service
2. Group of community-dwelling older adults aged 60 and over years from an Age UK strength and balance falls exercise group

The teleconferencing involved using Skype on the smartphones of health professionals and patients and connecting the phones to either a screen or TV. We know that older adults feel more comfortable using technology they are familiar with [27], and therefore, we thought this would be more acceptable than specific teleconferencing equipment.

The health professional workshop was run by a researcher who was also an occupational therapist (OT) in a different falls team. The older adult workshop was run by the OT and the lead researcher for the project. In the workshops, we discussed the initial concept of the technology with an explanation of why we thought it was important (perceived usefulness), what we were trying to achieve, and how teleconferencing could work for rehabilitation. We then connected the phone to a large screen (as would have been done for delivery) and demonstrated to the whole group what patients and health professionals would need to do and what they could see. We asked for feedback on the concept and whether participants (health professionals and older adults) thought health professionals and patients would be able to use it (perceived ease of use). Discussions on stands for smartphones and equipment used (whether to use Chromecast or a high-definition multimedia interface [HDMI] cable to connect the phone to a TV or screen) were included. Notes were taken on the feedback provided.

Contact with older adults and health professionals was classed as PPI rather than formal research. Therefore, we only collected aggregate details on gender, ethnicity, and previous experience of technology for participants and gender and clinical background for health care professionals.

### Usability and Feasibility Testing

The research proposed in this stage was predominantly qualitative. This enables us to establish whether the technology is acceptable to patients and health professionals (qualitative methods) and assess its usability and feasibility in practice (technology testing) and to make improvements if required. The study was granted ethical approval by the North West Greater Manchester Central NHS Ethics Committee (integrated research application system: 205980, June 2016).

#### Sampling Principles and Procedures

Older adults at risk of falls (aged 50 years and above), identified through the current community falls rehabilitation services from one service in Manchester, were recruited, with the aim to recruit 20 participants. Participants were those who would usually be offered a home exercise program by the service and could be at any stage in their rehabilitation (eg, we wanted patients both at the start of their program and also further on in their program so that we could assess the feasibility of the delivery of most of the evidence-based program through teleconferencing). Older adults who were unable to follow instructions and those with severe visual or hearing impairment were excluded. At this point, there were no other exclusion criteria. The lead researcher provided technical support to patients and health professionals during the study period.

#### The Intervention

##### The Technology

For testing, we used Samsung Galaxy S4 phones and *pay as you go* sim cards and 4G networks, and where possible, we connected them to the patients’ Wi-Fi networks. Both the health professional and patient had a phone provided by us that they connected to either their own TV or a provided screen either using a HDMI cable or through Google Chromecast. When the device was used for teleconferencing, it could be placed in a docking station, which then connected to the television.

The technology was tested using either 4G-enabled phones or by providing broadband at patients’ homes. The broadband (where not already in place) was paid for and set up by the research team with no cost to the patient. The smartphones and docking station were provided by the research team, and compatible screens were also made available. They were also given a wireless headset to ensure they could hear each other during the videocall. We used Skype for both individual and group-based virtual home exercise in the patients’ own homes, with health professionals delivering the exercise program from their offices.

##### The Exercise

Patients were offered standard service for 2 weeks (to ensure safety) before usability testing. They were then offered the same evidence-based home exercise program that is delivered through standard service, but it was delivered through the technology virtually. Patients received additional contact (twice a week rather than once a week) during the testing period. The health professional delivered the evidence-based Otago exercises [28], with additional exercises from the evidence-based falls management exercise (FaME) program where appropriate [29].

The technology was used to deliver the following:

1. One-to-one home-based exercise twice over a period of 2 weeks for an hour through the smartphone system.
2. A group-based strength and balance program (2-3 patients) once over a period of 1 week for an hour through the smartphone system. The health professional was able to see all the patients, and the patients were able to see each other.

The researcher was present with the patients at the time of the exercise session and supported the patient to use the technology where required.

#### Measurements

##### Usability

This included recording issues the health professional and the older adults faced with regard to the technology throughout the testing period (issue-log or field notes).

We explored usability issues such as setting up and connecting the technology and accessing Skype, requirement for internet access or testing of 4G through mobile phone and whether teleconferencing would connect, and whether it was reliable through the use of 4G technology rather than Wi-Fi. The positioning of the technology for delivery of exercise both in the patients’ homes and at the offices of health professionals.

##### Feasibility

The size of the groups receiving the intervention (ie, the ideal number of patients) and the types of exercise that could be delivered through the smartphone system were considered.

##### Interviews and Focus Groups

Health professionals from 3 falls services in Manchester were recruited to participate in 3 focus groups following the testing period. We chose focus groups, as each group of health professionals was a team delivering a service together. The focus groups allowed them to discuss their experiences and “bounce off” each other, eliciting more experiences and rich data. All members of the staff (n=17) in each team were given study information by their team leader and asked if they were available for a focus group at their place of work.

The service involved in the testing gave direct feedback on their experiences of using the technology. The other two services received a demonstration of the technology and were asked to give their feedback based on a similar interview schedule (Table 1).

Older adults who participated took part in a one-to-one interview from their own homes. The questions in the interview and focus group schedules were based on FAll Repository for the design of Smart and sElf-adaptive Environments prolonging Independent livinG (FARSEEING) [30] consortium guidelines (a European-funded project that examined the design and implementation of technologies around falls) and the TAM [26]. The following key areas were explored in relation to the hardware (phone and setup) and teleconferencing (Skype): ease of use, adaption of use, reliability, choice, and control. We explored whether it was acceptable and feasible for patients to receive their program in this way and whether health professionals were willing to deliver this way, and preference for group or individual virtual exercise was also explored. Open-ended questions were designed to elicit a wide-ranging response.

**Table 1:** Interview and focus group schedule.

Questions			Acceptability	FARSEEING^a^ guideline	TAM^b^	Feasibility
**Older adults’ interview schedule**						
	What did you like or dislike about using a smartphone to exercise with the health professional?		✓^c^	Ease of use Adaption of use Reliability	Perceived ease of use	✓
	Were there any issues with using a smartphone to participate in your exercise sessions?		—^d^	Ease of use Adaption of use	Perceived ease of use	✓
	Were there any issues with space to do the exercises?		—	—	—	✓
	Did you feel safe?		—	Choice and control Reliability	—	✓
	How did it compare to the normal program delivered in person by the health professional?		✓	—	Perceived usefulness	—
	What did you think about exercising in a small group?		✓	—	Perceived usefulness	✓
	Were there any issues?		—	Ease of use Reliability	Perceived ease of use	✓
	Did you enjoy it?		✓	—	Perceived usefulness	—
	What did you enjoy or dislike?		✓	—	Perceived usefulness	—
	Would you exercise in a group without a health professional?		✓	—	Perceived usefulness	✓
	Did you prefer exercising one-to-one or in a group?		✓	—	Perceived usefulness	—
	How did you feel about being provided with broadband? (where applicable)		—	—	—	✓
**Health professionals’ focus group schedule**						
	**Teleconferencing and taking part in the exercises using a smartphone**					
		ALL^e^: What do you think about delivering exercise virtually?	✓	Reliability Choice and control	Perceived usefulness Perceived ease of use	✓
		What do you think the barriers or issues are?	—	Reliability Choice and control Ease of use Adaption of use	Perceived ease of use	✓
		What do you think the advantages are?	—	—	Perceived usefulness	—
		CFS^f^: How was your experience of delivering exercises virtually?	—	Reliability Choice and control Ease of use Adaption of use	Perceived ease of use Perceived usefulness	✓
		CFS: Were there any exercises that you could not deliver?	—	—	Perceived usefulness	✓
		CFS: Were you able to adapt the exercises?	—	—	Perceived usefulness	✓
		CFS: Did you feel that there were safety issues?	—	Reliability Choice and control	Perceived usefulness Perceived ease of use	✓
		CFS: Did you feel that patients were confident in carrying out exercises in this way?	—	—	Perceived usefulness	✓
		CFS: For which patient group do you feel that this intervention would be appropriate?	—	—	Perceived usefulness	✓
		CFS: Were there any issues with connecting the technology?	—	Ease of use	Perceived ease of use	✓
		CFS: Did you feel that you had enough technical support?	—	Ease of use	Perceived ease of use	✓
		CFS: Were there any issues with Wi-Fi access or reliability?	—	Reliability	—	✓
		CFS: Were there any issues with having enough space or room to deliver the exercises from your office?	—	—	—	✓
		CFS: Did you feel that patients were safe?	—	Reliability	Perceived usefulness	✓
		CFS: How did it compare to delivering your normal home exercise service?	✓	—	Perceived usefulness	✓
		ALL: What do you think about using technology to deliver exercise virtually to a small group?	✓	—	Perceived usefulness	✓
		CFS: Were there any issues with using a smartphone to deliver to small groups?	—	Ease of use Reliability	Perceived ease of use	✓
		CFS: Did you feel that it was beneficial to patients. If so, how?	✓	—	Perceived usefulness	—
		CFS: Were there any issues with delivering in this way?	—	Ease of use Reliability	Perceived ease of use	✓
		CFS: What did the patients think of it?	✓	—	Perceived usefulness	—
	**Overall**					
		Would you use a smartphone again or continue to use it if you could?	✓	—	Perceived usefulness	✓
		If not, why not and which parts of using a smartphone did you not like?	—	Ease of use	Perceived usefulness Perceived ease of use	✓
		What needs to be improved for using this system in your routine practice?	—	Ease of use	Perceived ease of use	✓

^a^FARSEEING: FAll Repository for the design of Smart and sElf-adaptive Environments prolonging Independent livinG

^b^TAM: technology acceptance model.

^c^✓: the question relates to that concept.

^d^—: the concept does not apply to the question.

^e^ALL: all teams were asked, including the ones given a demonstration.

^f^CFS: the identifier for the team who did the actual testing.

#### Analysis

Follow-up interviews with patients, focus group data with health professionals, and field notes were analyzed together using framework analysis [31]. This is a method of research that provides a clear structure for the coding. NVivo 11 qualitative data analysis software (QSR International) was used to manage the data. The validity of the analysis was checked by returning to the data once themes were identified and also through independent coding conducted by a second researcher on a sample of transcripts. Two researchers conducted discussions around the codes that emerged. This approach ensures rigor [32] by checking the coding of the data. Data from the issue-logs were collated, summarized, and coded within the qualitative data and used to provide triangulation for the focus group or interview data.

## Results

### Initial Consultation

Initial informal consultation with 3 services indicated that teleconferencing could aid delivery of rehabilitation, reduce the commute time of health care professionals and their chance of being caught up in traffic, and provide extra support to patients. Health professionals suggested that any intervention had to be carefully planned due to safety issues.

### PPI Workshops

Demographics of the older adults and health professionals in the workshops are reported in more depth in a previous study [33]. We recruited 5 health professionals, including 2 physiotherapists, 1 OT, 1 rehabilitation assistant, and 1 assistant practitioner. A total of 8 older adults were recruited, 6 of whom were female and all were White British. Two of the older adults participating in the workshop had previously used technology such as smartphones, tablets, or computers.

#### Health Professional Workshop

The workshop with health professionals found delivering exercise safely was the priority. Health professionals felt that a risk assessment of patients’ home environment would be required to ensure that it was safe to exercise and that the equipment was positioned correctly, for example, to ensure that the equipment was positioned where patients could access support during their exercises. They also felt that there were some challenges in delivering exercises through teleconferencing and that they may need to be adapted to be completed remotely.

Practitioners did not want to replace face-to-face consultations with only remote monitoring, as they felt that it is important to have personal contact with the patients. Health professionals felt that face-to-face contact enabled other issues to be identified (non-exercise related) and was also important to ensure that patients conduct exercises safely. They had no preference for the different types of stands or headsets for delivery.

#### Older Adults’ Workshop

Older people did not want to lose their face-to-face contact with health professionals completely and expressed the fear that use of technology could mean that patients would no longer get visits from a health professional. Some older adults stated that the health care professional is the only person they see all week. They thought that extra virtual sessions with health professionals could provide opportunities to reduce loneliness and isolation.

Some older adults stated that they would not like any technology within their homes; they felt that with the presence of technology, their homes would not feel like a home, and they also found the technology intimidating.

Older adults in the workshop had no preference for the different types of stands or headsets, and they were quite happy to wear the headsets; in fact, they quite liked the idea of doing so, as it brought back memories from working.

### Usability and Feasibility Study

A total of 7 patients (4 men) with a mean age of 77 years (range: 64-92) participated, and of these patients, 6 agreed to be interviewed; for one interview, the participant’s son was also present. Only 2 of the participants who took part already owned a smartphone. Only 2 of the patients already had Wi-Fi, and 1 agreed to let us install Wi-Fi to enable them to use teleconferencing. A total of 11 health professionals took part in the focus groups; 8 were women, 9 were physiotherapists, 1 was a nurse, and 1 was an OT (see the study by Hawley-Hague et al [33] for further demographics).

Data were summarized under barriers and facilitators, with 7 further subthemes. We have also linked themes to the theoretical framework (Table 2). Two overarching themes related to smartphone were established and are discussed in a separate paper where patients used a smartphone app (see the study by Hawley-Hague et al [33]). Some themes only occur for either patients or health professionals.

**Table 2:** Themes and subthemes from the interviews and focus groups.

Theme and subtheme		Theoretical framework	Quotes	
			Patients	Health care professionals
**Barriers**				
	Poor connectivity	Reliability Perceived usefulness Feasibility	“If you’re not here to rectify it, I wouldn’t know what to do, would I?” [Male, aged 92 years] “So last time we got at least ten minutes, that’s the most we ever got, wasn’t it?” [Female, aged 69 years]	“When it worked it was good, but I must say after that session where it overheated so many times, following that I thought what is it, are we going to have that again. So I was very relieved to get through a session where it went all the way through” [Female, physiotherapist, S1] “They drained when we were actually using it. So battery life wasn’t good enough to do the actual full exercise programme” [Female, occupational therapist, S1] “It was just unfortunate that it was that patient where the phone froze on numerous occasions…then it was the secondary kind of safety issue of it freezes in the middle of the session” [Female, physiotherapist, S1] “It didn’t work as well, connectivity...I don’t think you could do it with 4G really” [Female, physiotherapist, S1]
	Cost of connectivity	Feasibility Acceptability	“It’s the expense of getting the landline as well as getting the broadband...you've got to have broadband and we're going to charge you bom-bom-bom, whatever it is, I didn't like so I got rid of it” [Male, aged 82] “we’d still have to pay the rental...” [Female, aged 69]	—^a^
	Safety concerns	Feasibility Perceived usefulness Perceived ease of use	—	“I had some concerns also about safety...I’d have thought if we’re doing a longer term study you wouldn't be there, and some of the positioning that the equipment would be in wasn’t necessarily as safe for the patients...” [Female, physiotherapist, S1] “Because things like when we went to feet, we couldn’t see feet...Yeah. It was those things that I hadn’t anticipated until we actually tried it...things like you couldn’t see if they had matching black socks then.” [Female, physiotherapist, S1] “...and I was trying to move so that I could actually see what was important, but then to get two people doing that was quite tricky. I couldn’t move them around” [Female, physiotherapist, S1] “...it was very difficult to hear. Because at some points there were almost four people talking...the participants were talking and you were kind of explaining to them” [Female, physiotherapist, S1]
	Intrusiveness of the technology	Adaption of use Feasibility Acceptability	“To leave something permanent it's got to have its place like the television” [Male, aged 82]	“I think the other thing that frightened the patients was the amount of equipment that came in, like the screens and the cables and that sort of thing. It is kind of intrusive into a person’s property” [Female, physiotherapist, S1]
	Group teleconferencing	Feasibility Acceptability	“I think you've got to be a certain type of person to do a group thing and I'm not that type of person actually, I just prefer to do it my way, my time, when I want, because if you're doing it with a group you're tied to however many number's in the group” [Male, aged 82] “Probably on my own to be honest but I was willing to give it a go testing that technology” [Male, aged 64]	—
**Facilitators**				
	Increased support or monitoring	Perceived usefulness Acceptability	“Oh yeah, it's nice...yeah, it's like being at a group” [Male, aged 74]	“If you deliver a one-to-one on the screen, that’s a fantastic idea. And I think it would relieve our time, the patient’s time. I think you’re kind of creating space...when we go and do a one to one at someone’s house you’ve got travelling time, you’ve got time in the house” [Male, physiotherapist, S2] “More reinforcement, isn’t it? So that’s good...monitor their adherence to the programme. Potentially less clinician time.” [Female, physiotherapist, S3] “you could phone them in or teleconference in if you like in between times and check, because you’ve already shown them, but you could check and do some basic correction and stuff in between and then go for your visits back to increase the programme...Ideally you would go back a few more times to really make sure their technique’s perfect, but if I feel they’re managing okay, they understand, they’ve got instructions and papers and all that type of stuff then I will let them go for a few weeks and then go back and see them.” [Female, physiotherapist, S2] “We often find on discharge that we’ve had voluntary drivers to bring them to groups and things, but then that’s not available on discharge; so people that would happily come out can no longer come out. So they would love to carry on exercising in a group, so they would fit into that criteria” [Female, physiotherapist, S3] “I think one of my chaps did. Because when his son was there in the house, oh, I don’t know anyone with Skype, well, I do, Dad, here we go…” [Female, physiotherapist, S1]
	Positive solutions	Feasibility Ease of use	—	“Like a CCTV camera, rotation...At least 180 degrees, and up and down” [Female, physiotherapist, S1] “You have to make sure that technique’s right, don’t you? That’s the thing” [Male, physiotherapist, S3] “I think if they’re screened properly and you’re checking them, and also if you’ve given them a certain exercise you’re confident with and then you go back to give them the next ones then I suppose they’re just as safe as if...because they’d be doing them by themselves anyway” [Male, physiotherapist, S3]

^a^—: the theme did not occur.

#### Barriers

##### Poor Connectivity

The reliability of teleconferencing was a very important issue, and reliability was threatened by a number of issues related to the connectivity of the phone during teleconferencing. One of the issues that occurred was overheating of the device during teleconferencing, which occurred mostly when testing the phone over 3/4G networks. This issue caused anxiety in health professionals. The patients were not as concerned as the health professionals, as a member of the research team was with them; however, they discussed the implications of what they would do if they were alone.

The battery life also seemed to be poor, and we think this was related to overheating due to poor connectivity. When the phones did not overheat, they froze during the teleconferencing, and this also caused concern, particularly for patient safety.

The lack of connectivity did not only cause the phone to overheat or freeze but also caused Skype sessions to suddenly switch off. We tested other forms of teleconferencing, such as Google Hangouts and WhatsApp video calling, but these performed more poorly in places with poor connectivity. In one female patient’s house, the reception was very poor, and we never managed to get to the end of a full rehabilitation session without the phone being frozen or the Skype session being disconnected.

##### Cost of Connectivity

As part of the study, we offered to fund broadband connections if needed. When exploring broadband as a solution to connectivity issues, we learned that a large number of patients do not have landlines; therefore, we could not provide broadband connections for these patients without disruption. In some cases, there was resistance to broadband even when we offered to provide it because of the cost that would need to be sustained once the study had finished. One patient previously had broadband but stopped it because of the cost. Some patients had their landlines taken out due to extra cost because they had mobile phones (even if not smartphones).

##### Safety Concerns

We have already outlined how poor connectivity caused issues with teleconferencing and concerns over safety. However, there were other practical safety issues around delivering teleconferencing through the phone.

There were concerns over the positioning of the equipment. The majority of patients conducted their exercises in their kitchen (using the kitchen worktop for support), and sometimes, they faced issues with finding enough space and room. We could not use their TVs as originally planned and had to use a separate screen. We used the built-in cameras of the smartphones and found that placing the phone on top of the refrigerator often gave the best view. However, placing the phone on the refrigerator caused other safety issues and would have required patients with balance issues to reach up in the absence of the researcher.

There were issues not only with positioning but also with view and contrast. If patients wore black trousers and black shoes, it was difficult to see their feet, and the room’s source of light also affected the view and what the health professional could see. This became a significant issue with group teleconferencing, as the picture of each person became smaller with more people on the screen. Issues with sound were also observed when we tested group teleconferencing (2 patients and the health professional). This was exacerbated by the time lag in Skype and led to patients talking over each other. These were the issues that were predominantly highlighted by health professionals and did not seem to concern patients.

##### Intrusiveness of the Technology

In addition to positioning and safety, there were issues with the intrusiveness of the technology. Originally, we wanted to use the patients’ own TVs for teleconferencing, but as most patients conducted their exercises in the kitchen, this was not feasible; therefore, screens were provided. The intrusiveness of the equipment was raised as an issue by the health professionals. One patient also discussed the worry that the equipment could be seen from his front window and that it could cause a risk of a break-in. He felt that participants had to feel that the equipment had a specific place for it not to be intrusive. However, most patients did not mind having the equipment in their house. Health professionals stated that this could become an issue if we tested it with more patients for a longer period.

##### Group Teleconferencing

Only 2 patients were able to take part in group teleconferencing, as we needed 2 patients with broadband to be recruited at the same time for it to be reliable. We tried group teleconferencing using 3/4G, which would not connect and thus was not feasible. However, we did discuss group teleconferencing with all patients who participated. Some of the patients felt that they were not “group people,” whether the group met face-to-face or virtually, although all participants agreed to test it for us. There were increased safety issues related to group teleconferencing in terms of view and sound. The service we worked with did offer group sessions, and only 2 of the patients recruited for the testing chose to attend a face-to-face group as well as perform their exercises at home.

#### Facilitators

##### Increased Support or Monitoring

Health professionals saw the idea of teleconferencing as a time-saving intervention with the potential to save travel time, which enabled them to invest that time back into patients. They also saw it as another tool to enable them to monitor patients’ adherence to their program and give them more support. During the follow-up phase of rehabilitation (where the health professional did not see the patient every week), they felt that the technology would enable them to give more input than a telephone call, allowing them to check technique. It would also enable the health professional to check up on the patients remotely and then see them face-to-face if required.

Group teleconferencing also provided an opportunity for group support that patients would not normally get when based at home. From the 2 patients who took part in the group teleconferencing, we received positive feedback, despite one being uncertain about groups. Despite their initial anxiety, this patient enjoyed the group sessions and went on to actually attend a face-to-face group exercise class. Health professionals could see the potential benefits of group teleconferencing for tackling social isolation and building confidence to attend a face-to-face group session. They also discussed how group teleconferencing could provide support through follow-on opportunities where transport was prohibitive to attending face-to-face follow-on groups. Teleconferencing provided an opportunity for other support as several patients went on to explore options for skyping family and friends.

##### Positive Solutions

Health professionals came up with active solutions for issues with teleconferencing, such as positioning of the equipment and the view of patients. They suggested getting cameras that could rotate so that the patient would not have to reposition them.

They discussed the types of patients it would work with, and that it would be important to ensure that technique was right face-to-face first before delivering virtual support and checking technique. If patients were given the right combination of support (a mix of face-to-face and virtual), they would not perceive a safety issue.

## Discussion

### Principal Findings

Using teleconferencing for the delivery of rehabilitation exercises for falls prevention seemed to offer more barriers than facilitators. However, the barriers are not insurmountable if we have better connectivity and equipment. The original aim was to make teleconferencing accessible and easy to use by using existing equipment (eg, smartphones’ cameras). Although the current technology system is acceptable (perceived usefulness) to health professionals and patients and adequate for follow-up support calls, it is not adequate for the delivery of exercises (not easy to use, feasible, or reliable) [26]. Phones overheated, there was poor connectivity where there was no Wi-Fi, and the view was not adequate for the delivery of new exercises. Issues highlighted around ease of use were predominantly related to the smartphone camera and positioning. The only issue raised with the software (Skype) was the view and sound during group teleconferencing, and the reliability of the teleconferencing was affected by connectivity regardless of the platform. Further equipment is required to enable the safe delivery of exercises to patients.

Overall, participants and health professionals in the workshops and usability testing could see some benefits of teleconferencing in terms of additional support that could be provided and better utility of resources, for example, travel time (perceived usefulness and acceptability). Increased contact and support was identified as the main facilitator for teleconferencing in both the workshops and usability testing and has been identified as important in previous studies [19], and it has also previously been found to lead to higher levels of adherence [15]. We know from other exercise studies and behavioral theory, such as the Theory of Planned Behavior, that social support or social norms (perception that the health professional thinks it is a good thing to do) from health professionals is important to exercise behavior [5,34].

Some of the older adults in the workshop and patients in the usability testing did have some concerns about bringing the technology in to their homes and the technology being intrusive (feasibility), which is something often found in the literature [27]. This was one of the reasons that we tried to focus on technology that patients would already have, for example, connecting phones to existing TVs. However, the location of the TV was not always the best place for the patient to exercise; therefore, separate screens were provided.

Patients who took part in the usability and feasibility testing at no point suggested that they would prefer face-to-face delivery or showed fear that technology would replace human interaction (acceptability and perceived usefulness), which is something often cited in the literature [27,35] and raised by older adults in the PPI workshop. Battery life was one of the other issues raised in the usability testing, and this was especially an issue when the phone was under high use (reliability). Battery life is a recurring issue in usability studies using smartphones [36,37]. The phones used have been upgraded for subsequent studies using smartphones.

In the usability and feasibility study, the main issue within the UK context was the lack of good 4G connectivity. This was particularly an issue in some of the more deprived areas of Manchester, where the connectivity was very poor (reliability and feasibility). It seems that due to socioeconomic reasons, patients had decided to have landlines removed and only used mobile phones (often not smartphones). This raises issues related to digital exclusion, an issue already associated with older adults and those who are on lower incomes [38]. In the current climate where rehabilitation is being delivered remotely because of the COVID 19 pandemic, there are concerns that patients will be excluded because they cannot afford Wi-Fi or a suitable device. They may be excluded because they do not have the skills to use the device even if they are provided with one (digital literacy), as our patients were provided with a large amount of support from the research team. They may have physical, cognitive, and sensory impairments or language barriers that make using technology challenging, particularly if they live alone [39].

Recruitment of health professionals covered 3 different teams in the workshops and usability and feasibility testing, but only 1 team used the technology in practice. We found that the teams that only had the technology demonstrated to them (service 2 and 3) but did not actually use it in practice were more positive about its use (perceived usefulness and acceptability) and generated further ideas around other functionality. In contrast, those who had used the technology (service one) identified more barriers, particularly because the technology was not reliable, but rather than being negative or resistive to technology, they also proposed potential solutions and implementation suggestions (adding another rotating camera).

### Limitations

There were limitations to the study during both the workshops and usability and feasibility study. During the workshop, we only illustrated how teleconferencing and the equipment would work, showing the patients and health professionals and older adults what would be seen. However, we did not demonstrate a full session. We did not ask health professionals to deliver an exercise session or ask the older adults to take part in one. This led to some issues not being identified until the usability testing that could have been preempted, such as the challenges related to the view from the phone camera.

At this point, the workshop was conducted with community-dwelling older adults and not patients; therefore, the participants were less frail and complex. It could be argued that they were two different populations, which may have influenced the feedback given. However, we would argue that it was a strength to represent a wide variety of older adults’ views. In both the workshops and the usability study, we had a good representation of gender across the older adults, patients, and health professionals.

For the usability and feasibility study, recruitment took longer than anticipated; therefore, a much smaller number of patients were recruited than initially planned. We were also unable to test group teleconferencing effectively, as only one set of patients had Wi-Fi at the same time. However, recruited participants represented a good mix of patients in terms of comorbidities, age, gender, and previous technology experience (some with experience of smartphones and some with no experience). None of the participants had previously used Skype or teleconferencing before the study.

The time period for testing the technology was short and may not have identified all the usability issues. If we had established a longer testing period, then we may have asked the patients to exercise alone using the technology without the presence of someone from the research team. However, during the testing period, we established that with the current technological setup, using the equipment alone would not have been safe.

### Conclusions

Overall, we established that teleconferencing as a way of delivering falls rehabilitation can be acceptable to this group of patients and health professionals if it works effectively. There is a lack of research on smartphone-based teleconferencing interventions for the delivery of falls prevention exercise programs.

A larger usability and feasibility testing study is required to establish whether better equipment for delivery improves usability and makes delivery more feasible. The intervention can only be effectively delivered in patients’ homes where there is Wi-Fi. The options for delivery still need further investigation, as it is clear from testing that in normal circumstances, teleconferencing cannot be used as a full alternative to face-to-face delivery and can only be used to reduce face-to-face visits and to enhance current care. This study provides important information to health professionals now having to deliver care remotely because of the COVID-19 pandemic. In its current form, although it could possibly be a suitable delivery method for some older adults (those who are able to conduct their exercises without the requirement of physical correction by the health professional) because of connectivity issues, it can only be a suitable option for some patients, not all. The intervention may work more effectively in other countries, such as in the Nordic countries where Wi-Fi is more widely available.

## Abbreviations

FaME: falls management exercise
FARSEEING: FAll Repository for the design of Smart and sElf-adaptive Environments prolonging Independent livinG
HDMI: high-definition multimedia interface
NHS: National Health Service
OT: occupational therapist
PPI: patient and public involvement
TAM: technology acceptance model
TV: television
